# Exploration of the Prognostic and Immunotherapeutic Value of B and T Lymphocyte Attenuator in Skin Cutaneous Melanoma

**DOI:** 10.3389/fonc.2020.592811

**Published:** 2021-02-26

**Authors:** Xubin Dong, Jingjing Song, Buran Chen, Yufeng Qi, Wenjie Jiang, Huihui Li, Danni Zheng, Yinghao Wang, Xiaohua Zhang, Haiguang Liu

**Affiliations:** ^1^ Department of Thyroid and Breast Surgery, The First Affiliated Hospital of Wenzhou Medical University, Wenzhou, China; ^2^ Department of Children’s Health Care, The Second Affiliated Hospital and Yuying Children’s Hospital of Wenzhou Medical University, Wenzhou, China

**Keywords:** B and T lymphocyte attenuator (BTLA), skin cutaneous melanoma, tumor-infiltration, tumor microenvironment, prognosis, immunotherapy

## Abstract

B and T lymphocyte attenuator (BTLA) is a newly identified immune checkpoint molecular belonging to the CD28 immunoglobulin superfamily. However, the expression and clinical value of BTLA in skin cutaneous melanoma (SKCM) has not been widely characterized. We found that BTLA levels were upregulated in metastatic melanoma compared to normal skin tissues and primary melanoma. Higher BTLA was also correlated with improved prognosis in SKCM based on several datasets. The multivariate Cox regression model revealed that BTLA was an independent survival indicator in metastatic melanoma. Tumor microenvironment analysis indicated BTLA was positively associated with the infiltrating levels of different immune cells and the activity of the anti-cancer immunity cycle. Importantly, BTLA accurately predicted the outcome of melanoma patients treated with MAGE-A3 blocker or first-line anti-PD-1. The present findings disclose that BTLA is a reliable biomarker for prognosis and immunotherapeutic response and might contribute to developing novel SKCM immunological treatment strategies.

## Introduction

Skin cutaneous melanoma (SKCM) is estimated to be the fourth leading component cause of cancer-related mortality worldwide (1). However, existing markers such as S100 protein (2), BRAF, and NRAS mutation (3) have hints on the diagnosis and prognosis of patients. Without early detection and primary treatment, metastatic SKCM becomes life-threatening. The efficacy of partial surgery and traditional chemotherapy has been limited, that it contributed to the production of targeted agents and immunotherapies (4). Checkpoint-modulating agents targeting programmed death receptor-1 (PD-1) and its associated ligand (PD-L1) and cytotoxic T-lymphocyte antigen-4 (CTLA-4) have been used as monotherapy or combination therapy monoclonal antibodies, showing long-term tumor containment and prolongation of survival (5). Immunotherapy drugs, such as Pembrolizumab (anti-PD-1 antibody), Nivolumab (anti-PD-1 antibody), and Ipilimumab (anti-CTLA-4 antibody), have played an important role in the treatment of advanced-stage melanoma since 2011 (6–8).

Although immunotherapy has increased the hope for advanced cutaneous melanoma to improve the prognosis of SKCM patients, most patients still show *de novo* or adaptive resistance, and multiple factors can influence the effectiveness of immune checkpoint blockade (9). The absence of tumor antigens (10), antigen presentation defects (11), mismatch repair deficiency (12), overall mutational load, neoantigen load  (13, 14), PD-L1 levels (15), intestinal microbiota (16), etc. influence immunotherapy effects. However, these factors are insufficient to achieve accurate prediction. Therefore, it is critical and challenging to explore other specific molecular characteristics to improve the prognostic and therapeutic effect.

B and T lymphocyte attenuator (BTLA) is the third inhibitory receptor expressed by T cells, B cells, natural killer (NK) cells, and antigen-presenting cells (17). BTLA is also a member of the CD28 immunoglobulin superfamily, which includes CTLA-4, CD28, inducible costimulatory molecule (ICOS), and PD-1 (18). Herpes virus entry mediator (HVEM) is the known ligand for BTLA in mice and humans, belonging to the tumor necrosis factor family. Ligation of BTLA by HVEM has been shown to recruit SHP-1 and SHP-2 protein tyrosine phosphatases, thereby suppressing T-cell receptor (TCR) activation (19). As a co-signaling protein, BTLA can regulate B cell receptor signaling, cytotoxic T-lymphocyte activity, and inflammatory cytokines production to suppress inflammatory responses (20–23). However, the clinical predicted value of BTLA has so far received little attention in SKCM research.

Herein, we comprehensively investigated several databases to reveal the expression pattern of BTLA and its association with the prognosis of SKCM patients. The gene enrichment functional process of BTLA further led to the analysis of the fractions of tumor-infiltrating immune cells (TIICs) in the tumor microenvironment (TME). Moreover, we explored the predicted value of BTLA in immunotherapeutic response. The present study aimed to reveal the expression pattern and potential prognostic and immunotherapeutic value of BTLA in melanoma.

## Materials and Methods

### Data Collection and Processing

We systematically searched for melanoma gene-expression profiles that were publicly accessed with full clinical annotations. Summarily, we gathered seven publicly available datasets to perform a series of analyses, including validation of the expression, inferring the composition of the TME, prediction of patient survival, and response to immunotherapy.

RNA-seq and corresponding clinical profiles for The Cancer Genome Atlas (TCGA)-SKCM samples were downloaded from the National Cancer Institute Genomic Data Commons (24). Since the TCGA-SKCM cohort only had one matched normal sample, we obtained 556 RNA-seq profiles of normal skin tissue from the Genotype-Tissue Expression (GTEx) dataset for a larger sample size (25). Batch effects were removed using the limma function normalizeBetweenArrays after combining transcriptomic data from TCGA and GTEx datasets. For TCGA and GTEx datasets, the expression profiles were transformed into log_2_ (Transcripts Per Million + 1) for downstream analyses. RNA-seq and sample profiles used for identification of BTLA expression and prognostic value [GSE65904 (26), GSE53118 (27), GSE98394 (28)], validation of BTLA predictive value in metastatic melanoma patients treated with the anti-PD-1 agent [GSE91061 (29) and Liu et al. (30)], and MAGE-A3 blocker [GSE35640 (31)] were obtained from the Gene Expression Omnibus (GEO) database. Among the TCGA-SKCM and GSE65904 cohorts, melanoma profiles were categorized into primary and metastatic subtypes based on official annotated information.

### Gene Expressional Analysis

TCGA-SKCM and GSE65904 cohorts included 471 and 204 cancer patients, respectively. Immunohistochemistry (IHC) images from the human protein atlas (32) were used for detecting subcellular localization and the distribution of BTLA comparing protein expression between SKCM and normal skin tissues. Immunohistochemical staining was performed using the same anti-BTLA antibody (Cat. No. HPA047211).

### Cell Culture, RNA Extraction, and qRT−PCR

Normal Human Epidermal Melanocytes, adult, the lightly pigmented donor (HEMa-LP) were cultured in Medium 254 (Cascade Biologics) supplemented with human melanocyte growth supplement (Cascade Biologics). Melanoma cell lines SK-MEL-28, LOXIMVI, and M21 were maintained in DMEM (Gibco, USA) containing 10% FBS (Gibco, USA) and 100 U/ml of penicillin/streptomycin (Invitrogen, USA). All culture was performed in a humidified incubator at 37°C and a 5% CO2 atmosphere.

Total RNA was extracted from cell lines using TRIzol reagent (Thermo Fisher Scientific, USA) in compliance with the manufacture’s specification. All RNA samples were temporarily stored at -80°C. The isolated RNA was measured at 260/280 nm to ensure the reliability of RNA quality and quantity. The range of 260/280 nm is among 1.86–2.05. The qRT-PCR reaction was performed using ReverTra Ace qPCR RT Kit (Toyobo, Japan) following the manufacturer’s protocol. The relative expressions of BTLA mRNA were calculated using the 2^-ΔΔCT^ method with GAPDH as an endogenous control. The primer sequences were used as follows: BTLA forward primer, 5’-AAGGACGAAATGGCAAGCAGACC-3’; BTLA reverse primer, 5’-AGGCAGCAGAACAGGCAGAAAC-3’; GAPDH forward primer, 5’-GTCTCCTCTGACTTCAACAGCG-3’; GAPDH reverse primer, 5’-ACCACCCTGTTGCTGTAGCCAA-3’.

### Survival Analysis

The outcomes of overall survival (OS), disease-free survival (DSS), and disease-free interval (DFI) were obtained from TCGA-Clinical Data Resource (CDR) (33). Kaplan-Meier curves with the log-rank test were performed by the “survival” package. Cancer patients with full clinical annotations were selected for the Cox regression analysis. Univariate and multivariable Cox regression models were built using the clinical parameters along with the respective BTLA expression data by the coxph function in the “survival” package. Only significant factors with a p-value of <0.05 in univariate analyses were included in multivariable analyses. The patients were divided into the low-risk group and the high-risk group following the median expression of BTLA.

### Functional and Pathway Enrichment Analysis

The correlation analysis was assessed by the function “cor.test” in R with the Spearman method. Genes that had highly ranked positive or negative correlation coefficients with BTLA were selected (Spearman correlation value > 0.4 or < −0.4, *p* < 0.001). “enrichGO” and “enrichKEGG” functions in the “clusterprofiler” package were used to perform Gene Ontology (GO), and Kyoto Encyclopedia of Genes and Genomes (KEGG) analyses, respectively. “ggplot2” and “enrichplot” packages were used for visualization.

### Immune-Related Analysis

ESTIMATE is a well-established algorithm to estimate immune and stromal fractions based on the RNA-seq profile (34). The ESTIMATE algorithm was used to quantify the immune and stromal proportions in the SKCM microenvironment. xCell is a novel and robust method based on ssGSEA (single-sample Gene Set Enrichment Analysis) that estimates the abundance scores of different cell types in the microenvironment. For cell-type specific analyses, the xCell algorithm was used to generate estimates for relative proportions of various immune cell types in the TME. For each cell type, xCell assigns enrichment scores across all samples by integrating ssGSEA and deconvolution methods (35). CIBERSORT is a support vector regression computational pipeline for the quantification of the tumor immune contexture from the human RNA-seq matrix (36, 37). Estimation of the total immune infiltrate in each sample, and immune cell subset was performed using the “CIBERSORT” package with the LM22 gene set. The absolute mode was referring, and quantile normalization was disabled using the RNA-seq TPM data (38).

For immune activity profiling, the tumor immunophenotype (TIP) pipeline was applied (39). This approach used a ssGSEA-based algorithm to evaluate the relative activity of the seven steps of the immune cycle, which is a process by which the immune system recognizes and kills cancer cells within bulk tumor samples. TIP scores for the TCGA-SKCM cohort were available in the online TIP server.

Tumor mutational burden (TMB) was calculated by the sum of the number of non-silent mutations based on the Mutation Annotation Format files (derived from Muct). Microsatellite Instability (MSI) status (MANTIS score) for the TCGA cohort was obtained from a previous publication

### Statistical Analysis

The Wilcoxon signed-rank test or Mann-Whitney U test was used to compare gene expression. Comparisons between multiple groups were conducted by Kruskal-Wallis one-way analysis of variance (ANOVA). The Mann-Whitney U test or Student’s *t*-test was used in two groups analysis. Spearman’s correlation coefficient ρ was used to measure the strength and direction of the association between two ranked variables. Survival analysis, functional enrichment analysis, and cancer-immunity-related analysis were performed in R version 4.0.0.

## Results

### The Expression Level of B and T Lymphocyte Attenuator

**Figure 1:**
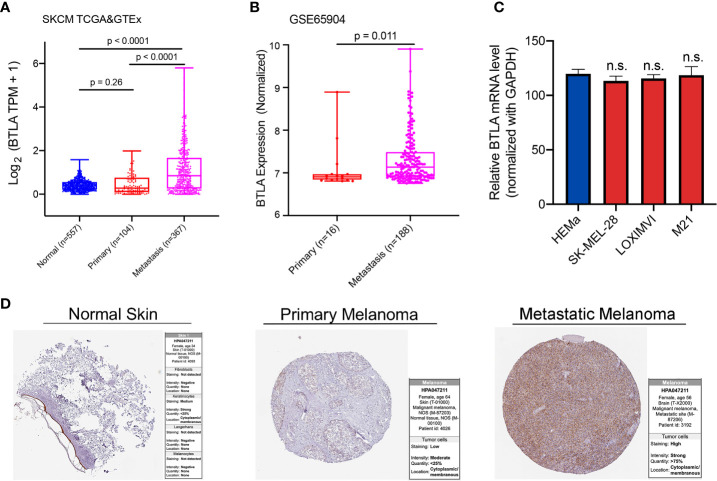
The expression of BTLA in SKCM. **(A)** BTLA mRNA levels in normal tissues, primary and metastatic SKCM tissues by TCGA and GTEx database. **(B)** BTLA mRNA expression levels in primary and metastatic SKCM tissues by GSE65904. **(C)** BTLA mRNA expression levels in HEMa and melanoma cell lines (SK-MEL-28, LOXIMVI, and M21). **(D)** BTLA protein expression levels in normal tissues, primary and metastatic SKCM tissues by the human protein atlas database. HEMa, normal human epidermal melanocytes; n.s., no significance. Statistical methods were as follows: **(A, B)** Wilcoxon rank-sum test.

Firstly, we analyzed BTLA expression using TCGA and GTEx RNA-Seq data. The expression of BTLA was higher in metastatic SKCM tissues than in normal and primary cancerous tissues (**Figure 1A**). To validate the aforementioned results, we used melanoma dataset GSE65904 to identify the differential expression of BTLA. Compared with primary cancerous tissues, the expression of BTLA was higher in metastatic SKCM tissues (**Figure 1B**). Moreover, we explored the level of LAGE3 in normal human epidermal melanocytes (HEMa-LP) and melanoma cell lines (SKMEL-28, LOXIMVI, and M21) by qRT-PCR. There was no difference in BTLA mRNA levels between melanoma cells and HEMa-LP cells (**Figure 1C**). The increases in metastatic SKCM tissues led to further evaluation of the protein levels of BTLA in SKCM. Analysis of BTLA protein patterns using IHC showed a higher expression in metastatic SKCM tissues compared to primary SKCM and normal tissues (**Figure 1D**).

### B and T Lymphocyte Attenuator mRNA Levels Predict Prognosis in Melanoma

**Figure 2:**
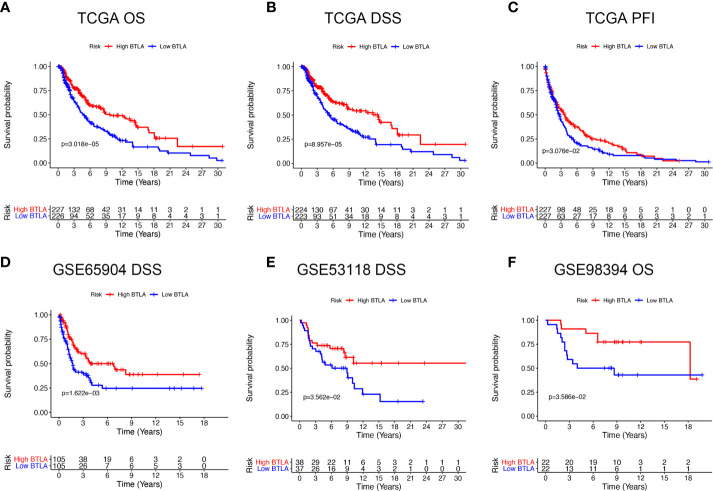
Kaplan-Meier survival curves comparing the BTLA^high^ and BTLA^low^ groups in SKCM in TCGA cohorts and GEO cohorts. **(A–C)** Survival curves of OS, DSS, DFI in TCGA SKCM cohorts. **(D, E)** Low BTLA expression was correlated with poor DSS in the GSE65904 and GSE53118 cohorts. **(F)** Low BTLA expression was correlated with poor DFI in the GSE98394 cohort. OS, overall survival; DSS, disease-specific survival; DFI, disease-free interval.

We first evaluated the influence of BTLA expression status on patient survival, including patients with primary and metastatic melanoma. Notably, the high BTLA group was markedly associated with long OS, DSS, and DFI (**Figures 2A–C**) compared to the low BTLA group. Additionally, we used different SKCM cohorts for validation. GSE65904 and GSE53118 showed that low BTLA expression markedly correlated with poor DSS (**Figures 2D, E**), and GSE98394 showed that low BTLA expression correlated with a poor OS (**Figure 2F**).

**Figure 3:**
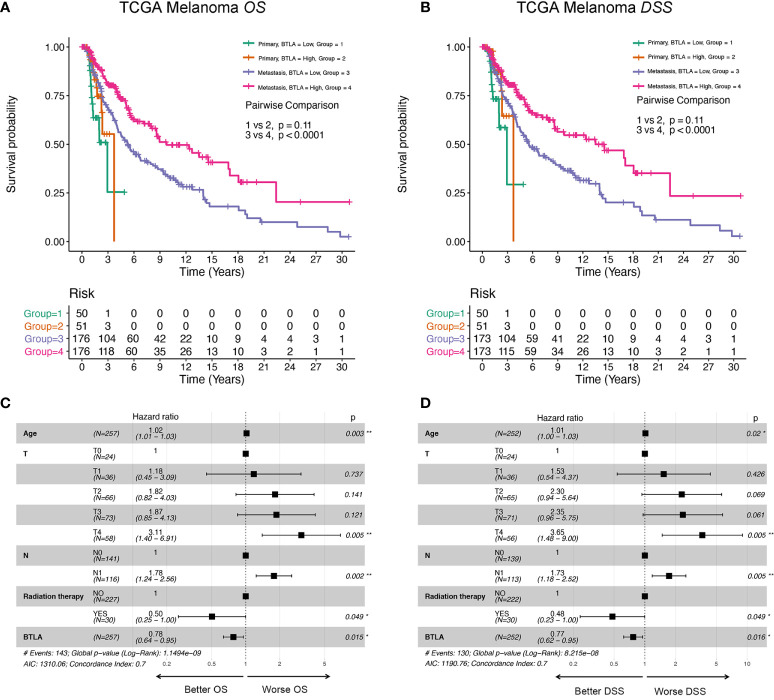
The survival curves and multivariate Cox analysis model of BTLA. **(A, B)** The OS and DSS curves using combinations of BTLA expression level and tumor subtypes. **(C, D)** The age, T, N, radiation therapy, and BTLA expression showed significance in multivariate Cox analysis forest graph of OS and DSS prognosis. OS, overall survival; DSS, disease-specific survival; T, tumor stage; N, regional lymph nodes metastasis stage.

Next, we analyzed whether the metastatic status was associated with the survival outcomes of BTLA expression level in melanoma. There was no significant association between BTLA expression and OS in primary melanoma, whereas high BTLA expression was associated with better OS in metastatic melanoma (**Figure 3A**). For DSS, the high BTLA group was correlated with a higher survival rate in metastatic melanoma but not primary melanoma (**Figure 3B**).

After deleting incomplete clinical samples, TCGA tumor tissues with clinical details were finally devoted to Cox regression analysis. In the univariate Cox regression model, age, tumor size, regional lymph nodes, radiation therapy, and BTLA expression were significant prognostic factors relevant to OS and DSS (*p* < 0.05, **Supplementary Tables 1**, **2**). Importantly, decreased BTLA expression markedly correlated with poorer OS (HR = 0.69, *p* < 0.001) and DSS (HR = 0.68, *p* < 0.001). In multivariable Cox regression, there were statistically significant differences between age, tumor size, regional lymph nodes metastasis, radiation therapy, and BTLA expression for OS and DSS prognosis (**Figures 3C, D**). These results suggested that BTLA was a potential prognostic predictor in metastatic melanoma.

### Functional Analysis and Predicted Signaling Pathways of B and T Lymphocyte Attenuator

**Figure 4:**
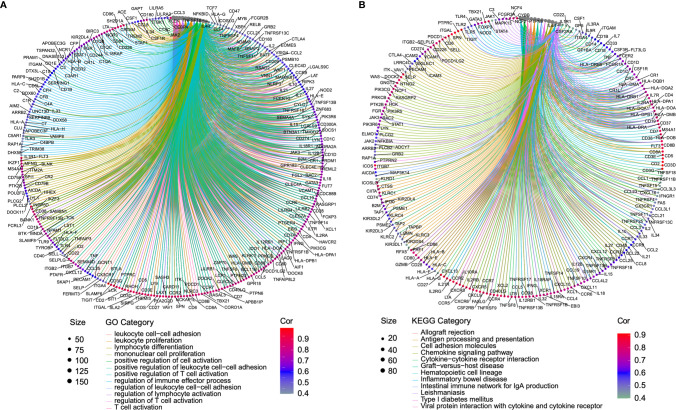
Function enrichment analysis of the co-expressed genes of BTLA. **(A)** The enrichment results of the GO. **(B)** The enrichment results of the KEGG signal pathway. Clusters are based on the gene count to measure the similarity between terms. The size of the gray circle represents the number of gene counts. Different color circles represent the correlation with BTLA. GO, the gene ontology; KEGG, Kyoto Encyclopedia of Genes and Genomes.

Genes co-regulated in a specific biological state is more likely to uncover the specific mechanisms [27]. Based on this, we selected genes with a strong co-expression correlation (Spearman’s correlation coefficient ρ > 0.5 or < −0.5, *p* < 0.001) with BTLA in the TCGA-SKCM cohort for functional enrichment analysis (**Supplementary Table 3**). Immune checkpoint genes such as TIGIT, ICOS, PD-1, and TIM-3 had a strong correlation with BTLA (ρ > 0.8). GO enrichment analysis showed that co-regulated genes almost mapped to GO terms related to immunity, such as leukocyte cell-cell adhesion, leukocyte proliferation, regulation of immune effector process, and regulation of T cell activation (**Figure 4A**, **Supplementary Table 4**). The KEGG enrichment analysis also displayed the enrichment of antigen processing and presenting, chemokine signaling pathway, and cytokine-cytokine receptor interaction (**Figure 4B**, **Supplementary Table 5**). The results demonstrated that BTLA plays an important role in the immune-related process in SKCM.

### Association Between B and T Lymphocyte Attenuator and Components of Tumor Microenvironment

**Figure 5:**
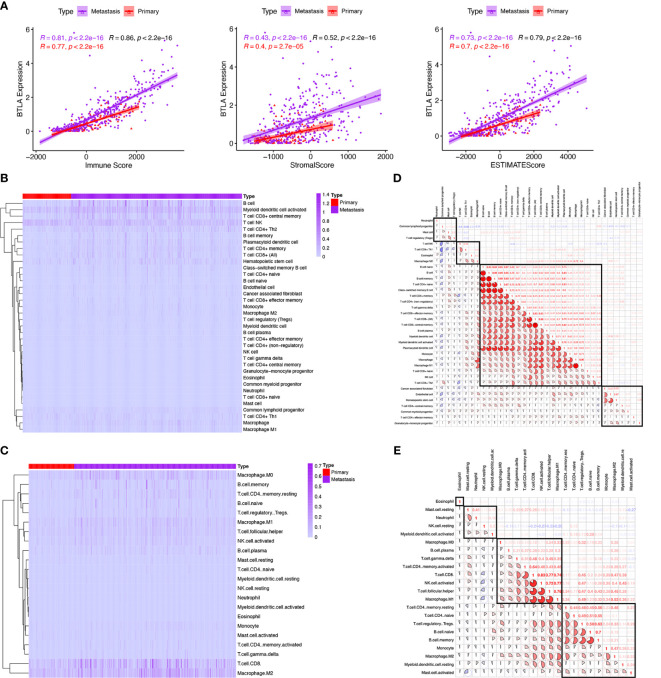
Comparison of tumor microenvironment scores with BTLA expression profiles and the relative proportion and correlation of tumor-infiltrating immune cells in SKCM. **(A)** Spearman correlations between BTLA and microenvironment scores calculated based on the ESTIMATE algorithm in primary and metastatic SKCM. **(B, C)** Heatmap illustrating the proportion of the immune cells in primary and metastatic SKCM microenvironment using xCell and CIBERSORT. **(D, E)** Correlation clustering of immune cells based on expression data to classify them into four groups by xCell and CIBERSORT.

TME is dominated by the tumor and various immune effector cells are recruited to the tumor site, largely in response to tumor-induced interactions (40). According to the aforementioned immune-related functional analysis and signaling pathways, we further investigated the TME in SKCM. We adopted the ESTIMATE algorithm to estimate abundances of cell types from the RNA-seq matrix. We found that BTLA expression in metastatic and primary SKCM positively correlated with immune score (Spearman’s correlation coefficient ρ = 0.81, *p* < 0.0001 and ρ = 0.77, *p* < 0.0001, respectively), stromal score (ρ = 0.43, *p* < 0.0001 and ρ = 0.40, *p* < 0.0001, respectively), and ESTIMATE score (ρ = 0.73, *p* < 0.0001 and ρ = 0.70, *p* < 0.0001, respectively) (**Figure 5A**). These results revealed that BTLA closely correlated with immune activation in the SKCM microenvironment.

### Relationship Between Tumor-Infiltrating Immune Cells in Skin Cutaneous Melanoma

TIICs are an integral component of the TME and correlate with prognosis and response to therapy (41). We used two different algorithms to reveal the significant differences among TIICs levels of primary and metastatic SKCM. Here, xCell, a gene set signature‐based method, was used for estimating the abundance of immune cell types from gene expression data. CIBERSORT was a versatile computational method that provided a score that could be compared between both samples and cell types. Of note, the landscapes of TIICs and tumor types are shown based on the CIBERSORT (**Figure 5B**) and xCell (**Figure 5C**).

Next, we divide TIICs into groups according to different methods based on all melanoma samples. In the gene-expression-based algorithm xCell, neutrophils were grouped with the common lymphoid progenitor, mast cells, and Tregs. NK T cells, type 1 T-helper (Th1) cells, eosinophils, and M2 macrophages were classified into one group. Fibroblasts, endothelial cells, hematopoietic stem cells, CD4+ central memory T-cells, common myeloid progenitors, CD4+ effector memory T-cells, and granulocyte-macrophage progenitors were categorized into another group. The remaining immune cell types were the major group, including native B cells, B cells, memory B cells, naive CD4+ T cells, class-switched memory B cells, CD4+ T cells, gamma delta T cells, CD8+ effector memory T-cells, CD8+ T cells, CD8+ central memory T-cells, plasma cells, plasmacytoid dendritic cells, monocyte, macrophage, M1 macrophage, CD8+ naive T-cells, NK cells and type 2 T-helper (Th2) cells (**Figure 5D**).

In the deconvoluting algorithm CIBERSORT, eosinophils were in a single group. Resting mast cells, neutrophils, resting NK cells, and activated myeloid dendrite cells were categorized into one group. M0 macrophages, M1 macrophages, B cells, delta gramma T cells, CD4+ memory activated T cells, CD8+ T cells, activated NK cells, and naive CD4+ T cells were classified into another group. CD4 memory resting T cells, naive CD4+ T cells, Tregs, naive B cells, memory B cells, monocytes, M2 macrophage, myeloid resting dendrite cells, and activated mast cells were classified into the last group (**Figure 5E**). These results revealed the potential close interaction between B cells, CD8+, and CD4+ T cells in the SKCM microenvironment.

### B and T Lymphocyte Attenuator Expression Levels Correlated With Immune Cell Infiltration

**Figure 6:**
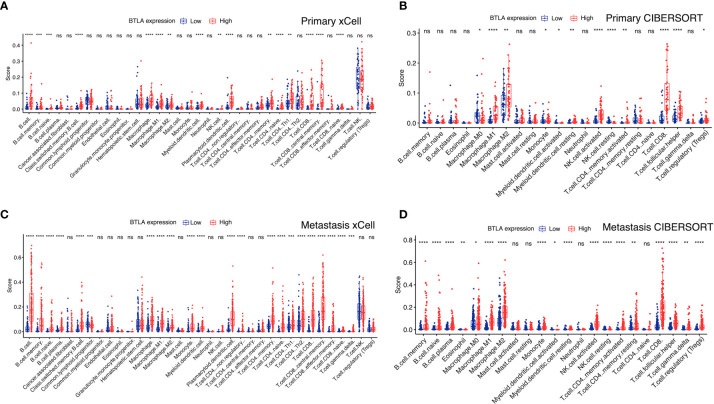
BTLA expression and tumor-infiltrating immune cells. Compare the immune infiltrated cell components quantified in **(A, B)** primary and **(C, D)** metastatic SKCM by algorithms xCell and CIBERSORT in the BTLA^high^ and BTLA^low^ groups. The scattered dots represent all score values, and the thick line represents the median value within each group. The bottom and top of the boxes are the 25th and 75th percentiles (interquartile range). The difference between BTLA^high^ and BTLA^low^ groups was compared through the two-sided Wilcoxon rank-sum test. ns: not significant, *p < 0.05, **p < 0.01, ***p < 0.001, ****p < 0.0001.

We combined xCell and CIBERSORT algorithms to investigate the immune infiltration level in primary and metastatic SKCM patients. In primary SKCM, M0 macrophage, M1 macrophage, M2 macrophage, myeloid activated dendrite cells, NK cells, and CD8+ T cells showed a higher proportion in the BTLA^high^ group than in the low BTLA^low^ group (**Figures 6A, B**). Moreover, the proportion of B cells, naive B cells, memory B cells, M0 macrophage, M1 macrophage, M2 macrophage, myeloid activated dendrite cells, CD4+ memory T cells, CD8+ T cells, and gamma delta T cells were significantly different between the BTLA^high^ and the BTLA^low^ patients with metastatic SKCM (**Figures 6C, D**). Therefore, combining previous studies and the aforementioned results, we concluded that the regulation of immune cells might be indicative of a potential mechanism in which BTLA was involved in SKCM.

### B and T Lymphocyte Attenuator Closely Related to the Cancer Immunity Cycle

**Figure 7:**
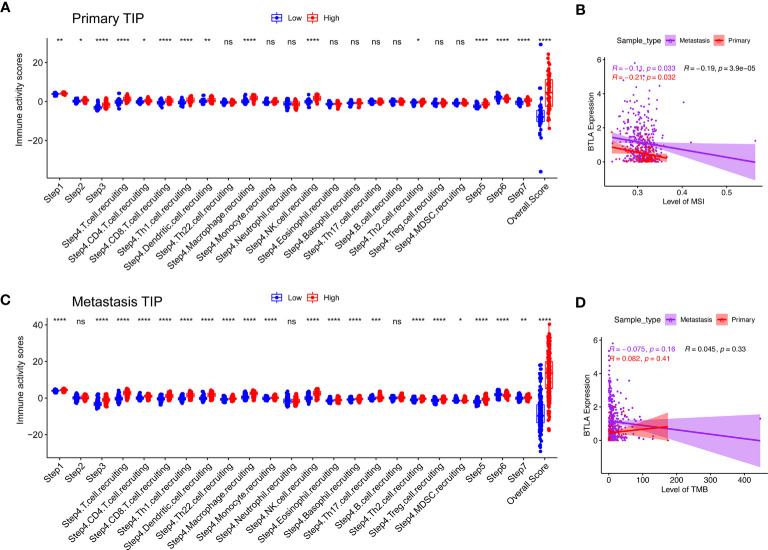
Compare the levels of cancer-immunity cycle, MSI and TMB between the different BTLA expressional groups. **(A, B)** Compare the levels of cancer-immunity cycle in primary and metastatic SKCM. **(C, D)** Compare the levels of MSI and TMB in primary and metastatic SKCM. TMB, tumor mutational burden; MSI, microsatellite instability. ns: not significant, *p < 0.05, **p < 0.01, ***p < 0.001, ****p < 0.0001.

The generation of immunity to cancer is a cyclic process leading to an accumulation of immune-stimulatory factors and amplification and broadening of T cell responses. These steps enable the anti-cancer immune response to kill cancer cells effectively, which prompted deepening the understanding of cancer immunotherapy (42). We used the TIP to estimate the activity scores of the cancer immunity cycle (**Figure 7**). After releasing cancer cell antigen and presenting cancer antigen, effector T cell responses against the cancer-specific antigens were primed and activated (steps 1 to 3). In both primary and metastatic SKCM samples, we observed that the BTLA^high^ group had higher anti-cancer immune scores in the trafficking of T cells to tumors (step 4), infiltration of T cells into tumors (step 5), killing of cancer cells (step 7), except the recognition of cancer cells by T cells (step 6). Recruiting of CD4+ T cells, CD8+ T cells, Th1 cells, dendritic cells, macrophage, NK cells, and Th2 cells had striking differences between the two groups in primary SKCM samples (*p* < 0.05). However, after including the changes in step 4, Th22 cells, monocytes, eosinophil, basophil, Th17 cells, Tregs, and myeloid-derived suppressor cells (MDSC) had higher anti-cancer immune scores in the BTLA^high^ group in metastatic melanoma. In summary, the BTLA^high^ group had a higher overall anti-cancer score in both primary and metastatic SKCM samples. It seemed that high BTLA expression led to more recruitment of T cells in SKCM, especially in metastatic SKCM. The critical balance between T effector cells and T regulatory cells was the key to the anti-cancer immunity cycle. Therefore, a BTLA^high^ state linked to an active anti-cancer immune response and BTLA may play a positive role in the cancer-immunity cycle.

TMB is an indicator of tumor mutation and an emerging biomarker associated with cancer prognosis (43). Similarly, evaluating MSI status could predict prognosis and guide immunotherapy (44). BTLA was weakly correlated with levels of MSI (**Figure 7C**, ρ = -0.14, *p* = 0.016) but not with levels of TMB (**Figure 7D**, ρ = −0.045, *p* = 0.33) in both primary and metastasis SKCM.

### B and T Lymphocyte Attenuator Predicted the Response of Immunotherapy

**Figure 8:**
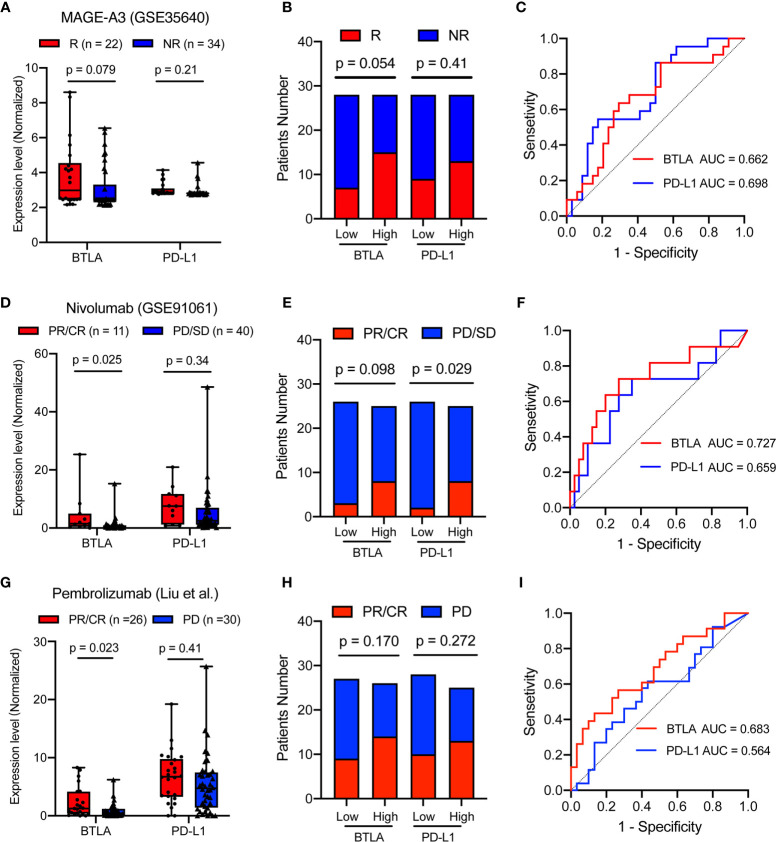
BTLA is predictive in immunotherapy response in advanced melanoma. **(A)** The comparison of the BTLA and PD-L1 expression between MAGE-A3 antigen vaccine therapy response groups. **(B)** The MAGE-A3 antigen vaccine therapy response in the different BTLA and PD-L1 expressional groups. **(C)** The predictive value of the BTLA and PD-L1 was measured by ROC curves in GSE35640 cohort. **(D)** The comparison of the BTLA and PD-L1 expression between anti-PD-1 immunotherapy response (Nivolumab) groups. **(E)** The Nivolumab immunotherapy response in the different BTLA and PD-L1 expressional groups. **(F)** The predictive value of the BTLA and PD-L1 was measured by ROC curves in GSE91061 cohort. **(G)** The comparison of the BTLA and PD-L1 expression between anti-PD-1 immunotherapy response (Pembrolizumab) groups. **(H)** The Pembrolizumab immunotherapy response in the different BTLA and PD-L1 expressional groups. **(I)** The predictive value of the BTLA and PD-L1 was measured by ROC curves in Liu et al. cohort. NR/R, non-response or response; PR/CR, partial or complete response; PD/SD, progressive or stable disease. ROC, receiver operating characteristic. Statistical methods were as follows: **(A, D, G)** Wilcoxon rank-sum test; **(B, E, H)** Two-sided Fisher exact test.

MAGE-A3 antigen vaccine therapies were developed for melanoma-specific immunization treatment (45, 46). Therefore, we investigated whether BTLA expression could predict metastatic patients’ response to MAGE-A3 immunotherapy. There was a trend that patients with lower BTLA expression were more likely to respond to MAGE-A3 (*p =* 0.079, **Figure 8A**). Compared with the BTLA^low^ group, the BTLA^high^ group showed a higher response rate (*p* = 0.054, **Figure 8B**). We further tested the prediction power of BTLA by calculating its AUC. The AUC was 0.662 and 0.698 using BTLA or PD-L1 expression as the only feature (**Figure 8C**). Both PD-L1 and BTLA show certain predictive value in MAGE-A3 therapy. These results suggested that BTLA was a significant predictor of immunotherapeutic response.

PD-L1 immunohistochemical expression in tumor tissues is the first clinically validated predictive biomarker and has been applied in clinical practice (47). In this study, we used PD-L1 gene expression as the IHC surrogate. Advanced melanoma patients, including those who had disease progression, resulted in a high rate of sustained tumor regression after receiving anti-PD-1 monoclonal immunotherapy (48, 49). We investigated the predictive role of BTLA in the Nivolumab immunotherapeutic effect. Patients with higher BTLA expression were more likely to respond to anti-PD-1 (*p* = 0.025), whereas the widely used PD-L1 expression showed no difference in immune response (*p* = 0.34, **Figure 8D**). Compared with the BTLA^low^ group, the BTLA^high^ group also showed a more than 2-fold anti-PD-1 response rate (*p* = 0.098, **Figure 8E**). Surprisingly, BTLA alone also enabled the classification of anti-PD-1 responders and non-responders with a considerably predictive power (AUC = 0.727) than the PD-L1 (AUC = 0.659, **Figure 8F**).

Moreover, we investigated the predictive role of BTLA in the Pembrolizumab immunotherapeutic effect (30). Patients with higher BTLA expression were more likely to respond to anti-PD-1 (*p* = 0.023), while the widely used PD-L1 expression showed no difference in immune response (*p* = 0.41, **Figure 8G**). Compared with the low group, neither the BTLA^high^ group nor PD-L1^high^ group showed a different response rate (*p* = 0.170 and 0.272, **Figure 8H**). Similarly, BTLA enabled the classification of anti-PD-1 responders and non-responders with a higher predictive power (AUC = 0.683) than the PD-L1 (AUC = 0.564, **Figure 8I**). These results indicated that BTLA might achieve better predictive performance than PD-L1 for anti-PD-1 therapies.

## Discussion

Despite advances in the understanding of tumor immune infiltrating cells and immunotherapy in recent years, poor clinical prognosis has been associated with *de novo* or adaptive resistance patients (50, 51). Therefore, there is an urgent need to find novel and effective immune-related therapeutic targets against SKCM. BTLA, also called CD272, was the third discovered molecule of the CD28 family after PD1 and CTLA-4 (17, 52). Studies have established the singular role of BTLA as both a co-stimulator and co-inhibitor to activated cancerous CD8+ T cells (53). A previous study revealed that BTLA detected in epithelial ovarian carcinoma (EOC) tissues can predict poor outcomes of patients, and BTLA inhibitor combined with chemotherapy could enhance immune activation and produce effective anti-tumor effects (54). Currently, phase I clinical trials NCT04278859 and NCT04137900 using recombinant humanized IgG4κ monoclonal antibody specific to BTLA are recruiting patients with an advanced unresectable solid tumor or a solid metastatic tumor. So far, the mechanism of BTLA in SKCM remains inconclusive. We aimed to explore the clinical value and potential biological roles of BTLA as an immunotherapy target in SKCM.

In our study, we found that BTLA was higher in SKCM tissues compared to normal tissues, whereas metastatic SKCM presented higher BTLA expression than the primary subtype. However, contrary to a previous study (54), upregulated BTLA correlated with favorable prognosis in TCGA cohorts and was verified by the GEO database. In the multivariable Cox regression model, we analyzed the clinical information in TCGA datasets to explore detailed mechanisms and the potential relationship of BTLA expression in SKCM. We found that BTLA expression was an independent factor of good prognosis. Although there have been some gene-signatures and predictive models in prognosis and prediction of response to immunotherapy in melanoma patients (55, 56), they are often composed of multiple genes and are not as good as a single gene in clinical practicality.

Our findings provide a novel understanding of cancer biology in SKCM and found that immune-related signal pathways were significantly correlated with BTLA. Moreover, immune-related genes such as TIGIT, ICOS, PD-1, and TIM-3 had a strong correlation with BTLA. The TME is essential to tumor initiation and progression, as well as the development of immune escape mechanisms (57). To investigate the BTLA immune regulating mechanisms in SKCM, we identified that the TME scores, including immune and stromal scores, were correlated with BTLA, particularly in metastatic SKCM. Besides, based on the aforementioned functional enrichment analysis of BTLA and its correlation with the TME, we concluded that BTLA expression was significantly associated with immune cell components in the SKCM microenvironment.

Because there is no gold standard for inferring immune infiltrate from the RNA-seq profile, we chose two algorithms to infer the composition of TIICs. We found that metastatic SKCM mediated higher immune infiltration levels of B cells, CD4+ T cells, CD8+ T cells, monocytes, M2 macrophages, and Tregs compared to primary SKCM. Moreover, the BTLA^high^ group demonstrated a higher immune infiltration level of M0, M1, M2 macrophage, and CD8+ T cells in both primary and metastatic SKCM tissues. Li et al. demonstrate that CD8+ T cells, previously defined as exhausted, are a highly proliferating, clonal, and dynamically differentiating cell population within the human SKCM microenvironment (58). The BTLA^high^ group recruited a high proportion of CD4+ T cells, CD8+ T cells, Th1 cells, dendritic cells, macrophage, NK cells, and Th2 cells in primary and metastatic SKCM tissues in the cancer immunity cycle. A durable and effective anti-tumor immune response requires the initiation and activation of CD8 + T cells towards effector CD8 + cytotoxic T lymphocytes (CTLs) (59, 60). These results indicated potential mechanisms where BTLA regulates macrophages, B cells, monocytes, and T cells, especially CD8+ T cells, in SKCM and maintains the homeostasis of the SKCM immune microenvironment. The independent prognostic prediction potential of BTLA in primary and metastatic SKCM patients may be related to alterations in the TME and TIICs.

Targeted immunotherapies have been increasingly used for better clinical outcomes. We attempted to investigate whether BTLA correlated with clinical outcomes in metastatic SKCM patients receiving immunotherapy. We found that BTLA was a significant predictor in predicting MAGE-A3 antigen and anti-PD-1 immune treatment response. The single-gene biomarker BTLA has potential clinical significance and transformability like PD-L1.In addition, a greater increase in CD8+ T cells in serial tumor samples during therapy correlated with a greater tumor-size decrease in imaging (Spearman’s correlation coefficient = −0.75; *p* = 0.0002) (60). The immune response to autologous tumors, including multiple T cell clones against multiple tumor antigens, is highly desirable and ideal (61). Taken together, these analyses suggested that BTLA might predict immune treatment response based on its function in balancing and regulating multiple immune infiltrating cells, especially CD8+ T cells, in SKCM. Thus, based on our analysis, we inferred that BTLA might be a positive stimulatory factor or an indispensable regulator in SKCM.

There are several limitations to our research. Firstly, this is a retrospective study and more tumor samples should be screened for cox regression analysis and immunotherapy response to verify the predictive performance as a biomarker in SKCM. Secondly, most of the existing TME computational methods are only limited to the transcriptional layer. Further analysis is necessary to quantify the particular biological processes and TME heterogeneity using multidimensional omics data. Lastly, our research revealed a markedly close interaction between BTLA and anti-cancer immunity. An elaborate regulatory mechanism of BTLA expression dysfunction in TME should be systematically elucidated *in vitro* and *in vivo*.

In summary, our study demonstrates that upregulated BTLA is associated with longer clinical survival, higher immune infiltration levels, and better immunotherapeutic benefit in melanoma patients. The present study provides insight into the role of the BTLA in anti-cancer immunity that may lead to the development of BTLA targeting therapy to assess the efficacy and receptiveness in SKCM treatment.

## Data Availability Statement

The original contributions generated for this study are included in the article/**Supplementary Material**, further inquiries can be directed to the corresponding authors.

## Author Contributions

XD contributed to the study design and bioinformatic analysis. JS contributed to the manuscript draft. BC, YQ, and WJ contributed to the review and edit. HuL contributed to the software analysis. DZ and YW contributed to the validation. XZ and HaL contributed to the supervision and methodology. All authors contributed to the article and approved the submitted version.

## Funding

This study was funded by the Natural Science Foundation of Zhejiang province (GF18H160072) and Wenzhou Science and Technology Planning Project (Y20190204).

## Conflict of Interest

The authors declare that the research was conducted in the absence of any commercial or financial relationships that could be construed as a potential conflict of interest.

## Supplementary Material

Supplementary Table 1 Univariate Cox regression for the overall survival rate of melanoma patients.

Supplementary Table 2 Univariate Cox regression for the disease-specific survival rate of melanoma patients.

Supplementary Table 3 Correlated genes of BTLA in TCGA melanoma dataset.

Supplementary Table 4 The gene ontology enrichment analysis of BTLA relevant signature genes.

Supplementary Table 5 The Kyoto Encyclopedia of Genes and Genomes enrichment analysis of BTLA relevant signature genes.
